# INTERDISCIPLINARY APPROACHES TO EPIDEMIOLOGY AND PREVENTION OF FALLS IN OLDER ADULTS

**DOI:** 10.1093/geroni/igad104.0780

**Published:** 2023-12-21

**Authors:** Wenjun Li, Wenjun Li

## Abstract

Prevention of falls is important to independent living and good health in older age. This symposium brings together five interdisciplinary studies on falls among community-dwelling older adults. The first presentation examines the relationship between multisite pain and fear of falling among older adults with dementia. The second discusses the differential effects of anxiety on indoor vs. outdoor falls. The cohort study found that anxiety was associated with higher rate of indoor but not outdoor falls. The third investigates racial differences in association of accelerometer measured physical activity with indoor and outdoor falls. The study found that a 30-minutes increase in moderate-to-vigorous intensity physical activity was associated with 25% lower rate of indoor falls and 42% higher rate of outdoor falls among non-White but not White participants, an interesting example of effect modification by race and location of falls. The fourth reports findings of a cross-sectional survey of older adults in Thailand which observed high prevalence of falls, and substantial differences in fear of falling, daily activities and quality of life between fallers and non-fallers, and between persons with and without fear of falling. The fifth reports rural-urban differences in circumstances and correlates of falls from the same study in Thailand. The study found higher fall rates, higher level of fear of falling and worse general health in urban than rural residents, highlighting the need for considering urban-rural differences in falls prevention. Together, these studies demonstrate the complexity and promises of interdisciplinary approaches to falls research.

